# A New Method for Modulation, Control and Power Boosting in Microbial Fuel Cells†

**DOI:** 10.1002/fuce.201800009

**Published:** 2018-06-13

**Authors:** I. A. Ieropoulos, J. You, I. Gajda, J. Greenman

**Affiliations:** ^1^ University of the West of England Bristol BioEnergy Centre Bristol Robotics Laboratory T‐Block, Frenchay Campus BS16 1QY Bristol UK

**Keywords:** Additional Electrodes, Microbial Fuel Cells, 3^rd^ and 4^th^ Pins, Redox Bias, Signal Modulation

## Abstract

Microbial fuel cells (MFCs) are energy transducers, which through the metabolic reactions of facultative anaerobic microorganisms, transform the energy in organic matter directly into electricity. Extrinsic parameters such as hydraulic retention time, fuel quality (type and concentration) and physicochemical environment of electrodes and biofilms (e.g., temperature, pH, salinity, and redox), can all influence system efficiency. This work proposes that MFCs can be “fine‐tuned” by adjustment of any of the physicochemical conditions including redox potential; in this context, an entirely novel method was investigated as a practical means of tuning, modulating and monitoring the redox potential within the electrode chambers. The method uses additional electrodes – known as 3^rd^ and 4^th^‐pins for anode and cathode chambers, respectively – which can be used in individual units, modules, cascades or stacks, for optimising the production of a large variety of chemicals, as well as biomass, water and power. The results have shown that the power output modulation resulted in an up to 79% and 33% increase, when connected *via* 3^rd^ and 4^th^ pins, respectively. Apart from power improvement, this study also demonstrated a method of open circuit potential (OCP) sensing, by using the same additional electrodes to both monitor and control the MFC signal in real time.

## 1 Introduction

By definition, a microbial fuel cell (MFC) is a system which converts microbial (bio‐chemical) energy (sometimes called “reducing power”) directly into electricity 1. It has been described as a bio‐battery that never runs out, provided that the microbes are kept fed. The feedstock (fuel) can be almost any soluble or particulate organic matter, including too‐wet‐to‐burn waste material (e.g., sludge) of which there is no shortage across the planet 2. This renders the MFC technology competitive either for waste utilization *via* energy recovery or, for microgeneration of electricity in diverse locations without conventional sources of electricity (whilst also re‐cycling waste) 3.

A “Platform Technology” (see Table 1) is one that can use the same fundamental system or base technology to drive a wide range of functions, applications or technologies across various sectors of the economy 4. Primary sectors of multiple applications include many industries whose main function is to extract resources or make raw materials (e.g., coal, oil, water, minerals, agricultural produce) so that the secondary industries can process these into manufactured goods and products. MFCs may take wastewater as the raw material (which is renewable and ever‐available from nature), and reduce the biological oxygen demand (BOD, i.e., clean it up) 5 whilst producing electrical power 6. For MFCs, many applications are possible across secondary industrial sectors especially in biotechnology and biological fuel cell industries, and with a modular stack system of highly controllable units, it is possible to envisage multiple outputs and thus, numerous emergent applications.

**Table 1:** Current examples where the MFC platform may fit in to bring forth better methods or even new technologies.

Sectors	Primary	Secondary	Tertiary	Quaternary
	Energy & resource recovery – water re‐cycling; mineral extraction	Light and heavy industries: chemicals, medical/pharma, food, paper/pulp, biofuels, wastewater treatment, bioreactors	MFC stack design, manufacturing, repair & maintenance	IT, robotics, electronics, A‐Life, artificial intelligence (AI)
Role for MFC	YES e.g., green chemistry; bulk chemicals, fine chemicals	SOME e.g., biotechnologies: specific utilization and/or specific production of: biomass, proteins, enzymes, polymers etc. incl. GEM	INCREASINGLY e.g., future need for MFC “servicing” for those using the technology	POSSIBLY e.g., bioelectronics, biohybrid devices, living sensors, EcoBots

The general idea of MFCs has been communicated more than a century ago 7, and many workers have contributed their knowledge towards the scale‐up and implementation in practical systems 8. The microbial fuel cell consists of two chambers (anodic/cathodic) for housing the corresponding electrodes and an ion selective polymeric or ceramic membrane separating these half‐cells 9. Anodophilic species of microbes colonize the anode electrode surface to form a mature biofilm‐electrode which, if perfusable in continuous flow conditions, remains stable through time, whilst continuously exhibiting “utilization properties” dictated by the types and proportions of living species contained within the biofilm 10. The biofilm as a whole is capable of metabolising the carbon‐energy by anaerobic respiration (i.e., anaerobic oxidation) whereby electrons abstracted from the fuel (in the form of NADH) are transferred by direct conduction from within the cell interior to the anodic electrode and NADH gets re‐oxidised into NAD^+^. The final end products are cations, such as protons, electrons, carbon dioxide and new biomass (the progeny cells of the growing biofilm, which are continuously released and washed out of the anodic vessel by hydrodynamic flow) 11. However, a single MFC, independent of size or shape, can only produce electrical power at a low voltage (e.g., 0.5–0.6 V), so a collective of at least two or more MFC units, connected electrically is required to step‐up the low voltage output to levels which can be used to power devices and modules that usually require voltages well over 1 V 12. It has been demonstrated in the last decade that one method of successfully pursuing this direction is by miniaturization and multiplication of small scale MFCs into stacks, demonstrating feasibility in practical applications 13, 14. Scaling up is critical for the technology to be implemented in practice and identify a route to market, independent of size or volume. It is a fact that more than one unit will need to be connected together, in order to increase the voltage and current to operational levels. Connections can be in series (to increase voltage), parallel (to increase current ) or a combination of the two (voltage + current boost). However, scientific investigations and scale‐up studies suggested that MFC operation at high reactor volumes are complex and often challenged with higher internal losses. This work aims to look into the properties of anodic and cathodic half cells of small‐scale MFCs in order to explore novel ways of connecting individual units together, in a way that would facilitate efficient scale‐up, offer power improvement, stack control and on‐line monitoring of the redox system.

With regard to the key transformations at the cathode, then it has been established that cathodic potential (redox) and pH play an important role in the production of water, hydroxyl radicals, hydrogen peroxide and other reactive chemical species, and these provide the catholyte with strong disinfective powers, similar to peroxides or bleach 15. At the anode, some functions and properties can also be affected *via* redox and pH, which strongly influence the metabolic rate of the biofilm and therefore its subsequent growth rate and power output 10. However, some specific bio‐transformations depend more critically upon the types of microbial species used to colonize the electrodes as either a monoculture or mixed species microcosms, as well as the redox potential level in a given environment. The designer‐operator agent can choose the biological properties by including a range of appropriate microbial biofilm species (e.g., salt tolerant 16, acid tolerant, thermo tolerant 17) with additional properties required for the desired functions (e.g., hydrolytic capability or expression of a therapeutic protein or production of new biomass) as colonizing inoculants.

What is certain is that MFC can be “fine‐tuned” by adjustment of any of the physicochemical conditions including the type of feedstock, flow rate‐dilution rate (h^−1^), temperature, salinity, pH and redox.

In this study, one particular method was investigated in depth as a novel way to fine‐tune and modulate the redox within the electrode chambers. The method uses additional electrodes (known as 3^rd^ and 4^th^ pins for anode and cathode chambers, respectively), and they may be used to operate at the level of single MFC units, cascades, arrays, modules or stacks, for both control and monitoring of the system in part or as a whole, and thus optimize the production of a large variety of chemicals, including biomass, water and power. The idea is derived from control theory and classic electrochemistry and the pins were used as the bias points for modulating the redox potential of the anolyte or catholyte in real time, thus directly affecting the level of power output and also providing a means for real‐time redox value measurement, which is akin to electronic transistors. To the best of the authors' knowledge, this is the first time that such a technique (Patent no. WO2016120641A1) using additional electrodes for modulation and control has been reported.

## 2 Experimental

### 2.1 Two‐chamber MFC Design and Operation

The MFCs comprised two (anode and cathode) 25 mL chambers separated by cation exchange membranes (CMI‐7000, Membrane International Inc. USA). Each chamber was made of acrylic material with dimensions h = 6 cm, w = 5 cm, l = 1.5 cm and the surface area of each membrane was 30 cm^2^. They were assembled using rubber gaskets, 5 mm nylon studding, washers and nuts, and were sealed with a non‐toxic aquarium sealant (Wet Water Sticky Stuff, Acquatrix, Witham, Essex, UK). For anodes and cathodes, plain carbon fiber veil electrode (20 g m^−2^ carbon loading; PRF Composite Materials Poole, Dorset, UK) with a total surface area of 270 cm^2^ (w = 30 cm, l = 9 cm) were folded, in order to fit into the chambers. For inoculation and feeding, municipal wastewater and activated sludge were provided from Wessex Water Scientific Laboratory in Saltford, UK. All MFCs were inoculated with activated sludge, with a natural pH of 7.8, and hence no artificial pH buffering was required. The MFCs were fed with activated sludge and tryptone yeast extract in the background, with sodium acetate as the main carbon energy. All MFCs were operated in fed batch mode, supplied with feedstock once daily at the start of the day.

### 2.2 Connection/Configuration of Working Cells

**Figure 1:**
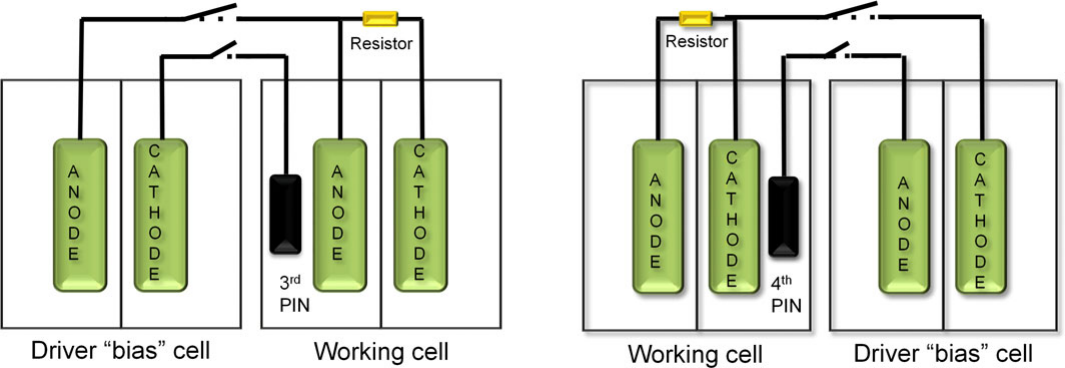
Left: Working and driver MFC circuit with 3^rd^ pin connection; Right: Working and driver MFC with 4^th^ pin connection.

For each experiment, two MFCs were used; one with additional smaller electrodes (pins) inside the anode and cathode (called the working MFC), and a standard 2‐electrode MFC as a driver. In the working MFCs, additional small electrodes (pins) with a size of 27 cm^2^ (1/10th of the size of the working electrodes) were inserted into the anodic (for single chamber open‐to‐air cathode types) and both anodic and cathodic chambers for all other experiments. The pin electrodes were made of the same 20 gsm carbon fiber veil material as for the standard anode and cathode electrodes. The pin electrodes were separated from the main electrodes by loose wrapping with an insulating plastic film (Parafilm^®^) in order to avoid direct physical contact, and consequently short‐circuit, in the same chamber. The driver MFCs did not have additional pin electrodes. The working and driver MFCs were connected *via* the additional pin electrodes. When the connection of two cells was ON (poise period), the anode of the driver MFC was connected to the anode of the working MFC, whereas the cathode of the driver MFC was connected to the 3^rd^ pin electrode. In the case of a 4^th^ pin, the cathode of the driver MFC was connected to the cathode of the working MFC, whereas the anode of the driver MFC was connected to the 4^th^ pin. The temporal connection of the driver to the working cell to poise the voltage of the working cell is shown in Figure 1.

### 2.3 Data Capture and Calculations of Power Output

The MFC output was recorded in real time as millivolts (mV) using an ADC‐24 A/D converter computer interface (Pico Technology Ltd., Cambridgeshire, UK). The current (*I*) in amperes (*A*) was determined using Ohm's law, *I* = *V*/*R*, where *V* is the measured voltage in volts (V) and *R* is the external resistive load value in ohms (Ω). Power (*P*) in watts (W) was calculated by multiplying voltage with current; *P* = *I* × *V*. Current density (*J*) and power density (*PD*) were calculated in terms of electrode total macro surface area; *J* = *I*/α and *PD* = *P*/α, where α is the total anode electrode surface area in square‐meters (m^2^). Internal resistance was calculated by applying Kirchoff's voltage law: R_INT_ = (V_O/C_/I_L_) – R_L_, where V_O/C_ is the open‐circuit of the MFC, I_L_ is the current under a load and R_L_ is the value of the load resistor.

### 2.4 Polarization Experiments

Cell polarisations were obtained by connecting a DR07 decade resistor box (ELC, France). Data were produced by varying the external resistance from 30 KΩ to 10 Ω at time intervals of 3 min after the MFCs had established a steady‐state open circuit voltage (OCV).

## 3 Results and Discussion

### 3.1 Power Boosting Effect

**Figure 2:**
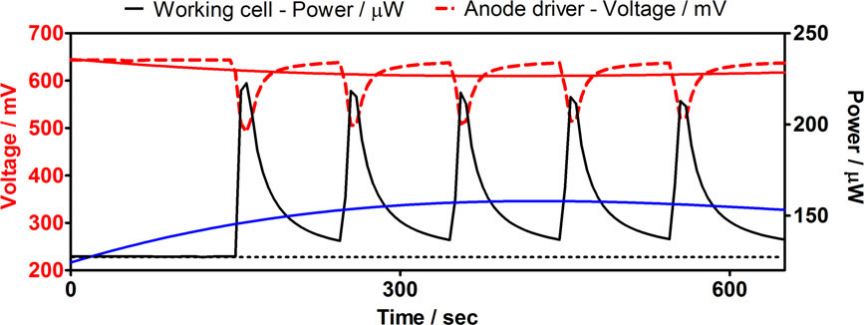
Power modulation of working MFCs (*n* = 3) through 3^rd^ pin bias connection. Data shown are the average for the three working (black solid line) and three driver MFCs (red dotted line). Duty cycle was 10 s ON, 90 s OFF. The black dashed line shows the constant power output that the working MFCs would have generated if not modulated. The blue solid line is a 2^nd^ order non‐linear regression curve, which shows how much – on average – the working MFCs' power output increased.

As mentioned above, the MFCs were complemented by the addition of an extra smaller electrode which would be used as the bias point for effecting modulation from an external source, i.e., another MFC. The smaller electrode added inside the anode has been termed “3^rd^ pin” to signify that it is the third electrode added inside the MFC. Figure 2 shows the power level modulation of one “*working MFC*” when a separate “*driver MFC*” was connected to its anode *via* the 3^rd^ pin. The external load of the driver MFC was disconnected (i.e., open circuit condition) when connected/disconnected to the pin electrode. In order to poise (voltage bias) the working MFCs, the working and driver MFCs were repeatedly connected for 10 s and then disconnected for 90 s (10:90 sec duty cycle), five times. When the two cells were connected (poised), the power output of the working MFCs increased (black solid line), whilst the voltage of the driver MFCs decreased (red dotted line). On average, the power output modulation resulted in a 72% increase (min: 65%; max: 79%), and this appeared to be reproducible without a deteriorating effect.

**Figure 3:**
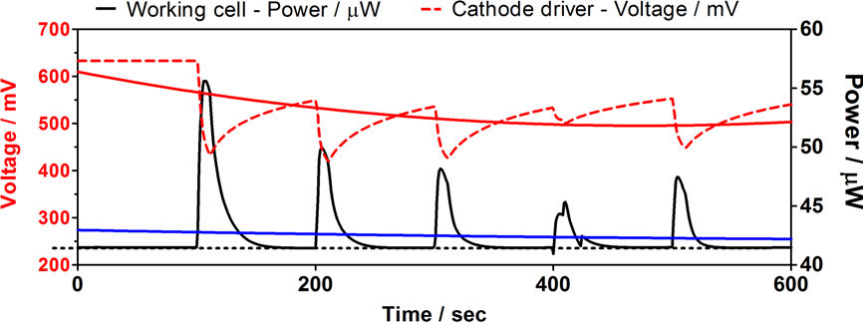
Power modulation of working MFCs (*n* = 3) through 4^th^ pin bias connection. Data shown are the average for the three working (black solid line) and three driver MFCs (red dotted line). Duty cycle was 10 s ON, 90 s OFF. The black dashed line shows the constant power output that the working MFCs would have generated if not modulated. The blue solid line is a 2^nd^ order non‐linear regression curve, which shows how much – on average – the working MFCs' power output increased.

A similar but slightly different effect was observed when a “*driver MFC*” was connected to a “*working MFC*” cathode, via the 4^th^ pin Fig. 3. In this particular case, the maximum power output recorded from the poising technique was 56 μW, which corresponds to a percentage increase of 33%, and the minimum was 45 μW, which is of the order of 7% increase. On average, power output increased by 17.5% and the level of power increase deteriorated with the number of modulation cycles. The voltage level of the “*driver MFC*” decreased in proportion to the increase in power of the “*working MFC*”. It is worth noting that the last cycle of modulation, resulted in a higher power output from the “*working MFC*”, compared to the previous one, which perhaps suggests that the “*working MFC*” was beginning to respond more positively to the poising action, and this is more in line with the “*working MFC*” behavior when modulating the performance *via* the 3^rd^ pin in the anode (Figure 2).

### 3.2 Real‐time monitoring of potential difference during operation (“dynamic open circuit potential”)

As mentioned above, the additional 3^rd^ and 4^th^ pin electrodes were not in direct contact with the working electrodes in the anode and cathode, respectively. This suggests that if a separate voltmeter is connected to the two pins, then in principle, the measured voltage difference should reflect the real redox potential difference value between the anolyte and the catholyte. In other words, it could be used as a real time voltage‐monitoring tool, even when the MFC's main anode and cathode electrodes are connected to a load i.e., producing work (power). This is of interest, since all the traditional methods for determining the internal resistance of MFCs require the measurement of the open circuit voltage, which is effectively the measurement of the two redox potential values in each of the half‐cells, but this traditionally requires the circuit to be interrupted. However, in the present case, it is suggested that the additional pins could be used to provide a real time monitoring capability for the MFC, whilst still producing power. In order to investigate this, working MFCs with 3^rd^ and 4^th^ pins were subjected to polarizsation by dynamically changing the external load, whereas a separate voltmeter was connected across the 3^rd^ and 4^th^ pin terminals in order to measure the real time MFC voltage behavior.

**Figure 4:**
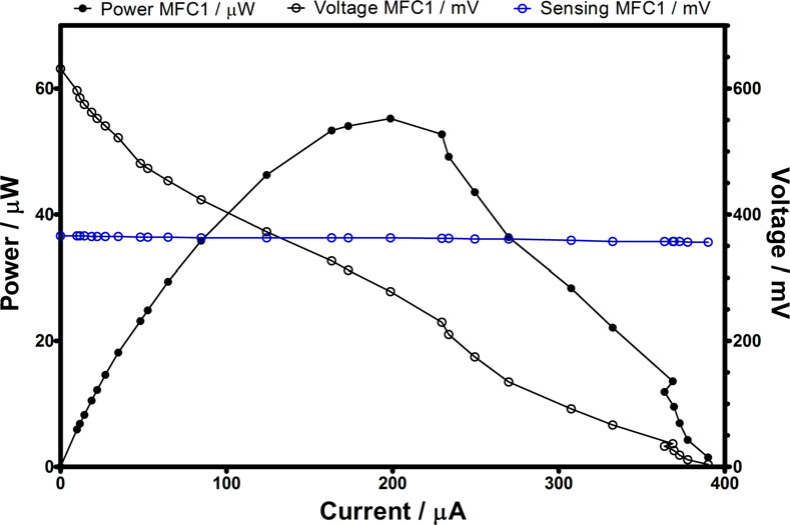
Pin electrodes performing open circuit potential “sensing” during polarization experiments. Black lines (open and closed symbols) represent the traditional polarization and power curves of a MFC, whereas the blue line represents the continuous monitoring of the working MFC's potential between the two half‐cells.

Figure 4 (blue line) shows that the potential difference between the 3^rd^ and 4^th^ pins remains constant throughout the polarization experiment and very close to half the value of the starting open circuit voltage. The stability of this measured signal implies that this is a mature and well performing MFC, as also shown from the power and polarization data and that this potential value can indeed be used for more accurately determining the internal resistance of the system, i.e., without having to use the initial, and quite possibly incorrect, value of open circuit voltage.

It is unknown how pin modulation is effective for MFCs within cascades or stacks, when the main (“*working*”) MFCs are connected in series and/or parallel as a collective. It is also unknown to what extent the pin material (carbon or various non‐corrosive metals of different electrochemical properties, such as Au, Ag, Pt, Ru, Rh, Pd, Ir and others) will affect the performance.

The mechanisms at play to explain the phenomena of modulation of the anode include (i) the establishment of a redox gradient within the fluidic interspace between the 3^rd^ and/or 4^th^ pin(s) and the anode (ii) an increase or decrease of redox around the biofilm electrode depending on the voltage supplied (iii) favouring the metabolism of low redox microbial respirators and/or high redox microbial respirators 18. It should be noted that in comparison with overpotential, the amount of energy required to maintain the voltage (provided by the “*driver MFC*”) is low because the resistance of the pathway from anode to 3^rd^ pin (and cathode to 4^th^ pin) is lower than the internal resistance of the whole MFC. The 3^rd^ and 4^th^ electrodes may thus be able to accomplish microbial electrolysis cell (MEC) like functions without the need of the (relatively) high levels of power required to accomplish conventional working electrode overpotential levels for electro‐fermentation or electro‐synthesis. It may also be possible for one “*driver MFC*” to modulate a plurality of “*working MFCs*”, and this is likely to be subject to solution conductivity, which will form part of our future work.

The finding that monitoring the output between the 3^rd^ and 4^th^ pins gave a voltage difference indicative of the redox difference between anodic and cathodic half‐cells and that this measurement will be related to the internal resistance of the working MFC, is novel and of particular interest for controlling the quality of electricity produced. Such readings may be taken at any time during the operation of the MFC, in contrast to the conventional method of calculating R_INT_ by recourse to open circuit measurements that interrupt the power.

A ‘Platform Technology' is one that can use the same fundamental system or base technology to drive a wide range of functions, applications or indeed spin‐off technologies. For MFCs, many applications (across industrial sectors) may use the same platform technology even though each unit, cascade or stack may be controlled or fine‐tuned (with the help of the 3^rd^ and 4^th^ electrodes) to drive a wide range of different applications or functions crossing a wide range of industrial sectors, including biomass, chemicals, water and power. The same basic modular design may serve for all.

## 4 Conclusions

A new connection method using additional pin electrodes provides not only improved power but also system modulation and OCP sensing. This novel connection through pins could make MFC systems more intelligent by enabling elaborate rapid control of the system, even at stack/cascade level through multiplexing. This may improve the modular “platform” approach with better fine‐tuning of conditions for increasing chosen bio‐transformations.
